# Female genital injury—which findings have to be considered physiological using colposcopy with and without toluidine blue dye?

**DOI:** 10.1007/s12024-021-00417-6

**Published:** 2021-10-06

**Authors:** Clara Berlit, Marc Sütterlin, Kathrin Yen, Christel Weiß, Sarah Heinze, Benjamin Tuschy, Sebastian Berlit

**Affiliations:** 1 Department of Obstetrics and Gynaecology, University Medical Centre Mannheim, Heidelberg University, Theodor-Kutzer-Ufer 1-3, 68167 Mannheim, Germany; 2 Institute for Forensic and Traffic Medicine, Heidelberg University, Heidelberg, Germany; 3 Department of Medical Statistics, Biomathematics and Information Processing, Medical Faculty Mannheim, Heidelberg University, Mannheim, Germany

**Keywords:** Genital injury, Colposcopy, Toluidine blue dye, Sexual assault, Menopausal status

## Abstract

This study aimed to assess the validity and efficacy of blue dye in colposcopic assessment of genital injury in pre- and postmenopausal women with and without history of consensual sexual intercourse. Two hundred women were prospectively enrolled and examined colposcopically with and without toluidine blue dye in order to detect and categorize genital lesions (laceration, bruise and abrasion). Examination of genital trauma was accomplished in a standardized way and findings were photo documented. A wide range of influencing factors with a potential impact on prevalence and nature of genital injury was recorded beforehand using a questionnaire. The frequency of diagnostic injury differed substantially depending on the examination technique, ranging from 9% using colposcopic magnification only to 28% with the additional use of toluidine blue dye. A vertical laceration affecting the posterior fourchette was the most frequent lesion detected (17%, n = 32). Menopausal status seems to have significant impact on genital injury prevalence (*p* = 0.0165), as 42% (16/ 38) of postmenopausal compared to 24% (36/ 151) of premenopausal women had at least one genital lesion. Furthermore, vaginal medication *(p* = 0.0369), vaginal dryness *(p* = 0.0228), dyspareunia (*p* = 0.0234) and low frequency of sexual intercourse (*p* = 0.0022) were found to significantly correlate with the presence of genital lesions. According to our findings, standardized colposcopy in combination with toluidine blue dye facilitates accurate assessment of genital lesions. Genital trauma situated at another site than the posterior part of the vaginal introitus seems to be uncommon after consensual intercourse.

## Introduction

Differentiation between genital injury due to consensual penetrative vaginal intercourse and genital injury caused by non-consensual penetrative vaginal intercourse is a matter of ongoing scientific debate and obviously incorporates a fundamental issue in sexual assault medicine.

The first hypothesis regarding this context rose in the mid-twentieth century as authors stated that physiologic changes (e.g. lubrication) and concurring interaction during consensual intercourse prevented genital trauma [1, 2]. On the other hand it was supposed that women who had been raped lacked these reactions, so that genital harm was possible. This theory of genital trauma constituting proof for sexual assault was only scrutinized decades later. The presumably most cited investigation in this context is by Slaughter et al. [3]; as one of the first studies to compare genital injury after consensual and non-consensual intercourse using magnification techniques authors found genital trauma in 11% of consenting women and in 68% of rape victims. With these results it was evident that consensual intercourse might also lead to genital injuries, which on the other hand does not have to be the case in women who allege sexual assault.

Until now further genital injury research was undertaken with the vast majority of investigations concentrating on non-consensually caused trauma; most data were generated retrospectively after reviewing sexual assault records. However, the existing literature lacks primarily methodological consistency leading to inconclusive results; injury rates vary from 4 to 89%[4]. The reasons for these differences are varying examination protocols, as some authors counted macroscopically detected injuries only, while others used colposcopy solely or in combination with toluidine blue dye, which presumably results in higher detection rates [4]. Furthermore, the definition of what constitutes a “genital injury” varies. While some studies included more ambiguous findings as “redness” and “swelling”, which are incorporated in the “TEARS” (Tear, Ecchymosis, Abrasion, Redness and Swelling) classification, others concentrated on lacerations only [4]. Given these inconsistencies the inevitable need for comparable data is obvious.

Only a few investigations assessing genital trauma after consensual intercourse exist. Just as in the non-consent-publications, these studies vary in injury definitions, study protocols and investigative techniques. Two reviews of the literature were undertaken with the aim of locating existing evidence concerning genital trauma after consensual sex [4, 5]. Due to the varying methodology in both publications it was concluded that existing data is hardly comparable. The more recent review by Astrup et al. includes 9 investigations of adult, sexually active premenopausal women [5]. The type of injury mostly detected was a single laceration in the posterior vaginal orifice (“6 o’clock position”), which was highly consistent between the investigations. Interestingly the authors found that in women who were recruited before sexual intercourse more injuries (prevalence ranging from 25 to 55%) were found compared to investigations in which women were recruited after (prevalence ranging from 4 to 11%) having had sex. Finally, smoking status and the penetrative use of fingers during intercourse were found to lead to a higher injury prevalence. On the other hand, parity, the use of tampons, lubricants and contraceptives seemed to not affect genital trauma.

Due to the inconsistency of the existing literature and the fact that to the best of our knowledge there exists no information on genital injury with and without history of consensual intercourse including postmenopausal women, we designed this investigation. We hence deliberately chose to recruit women without scheduled consenting sexual intercourse in advance, in order to analyze genital trauma in a “real world setting” assessing possible influencing factors.

## Materials and methods

This investigation was approved by the Ethics Committee II of the Medical Faculty Mannheim, Heidelberg University, Germany (2017-519 N-MA). A total of 200 women presenting at the Department of Gynaecology, University Medical Centre Mannheim between August 2017 and July 2018 were included in this prospective study after informed consent was obtained.

Exclusion criteria were pregnancy (including puerperium), age below 16, prior extensive genital surgery (e.g. vulvectomy, female genital mutilation) concomitant malign disease and/ or dermatological comorbidities (e.g. lichen sclerosus, lichen ruber planus, vulvar intraepithelial neoplasia). Written consent was obtained from all participants. Prior to the clinical investigation participating women were asked to fill in a questionnaire assessing behavioral and demographic parameters, which could potentially influence the appearance of their external genitals. Besides common demographic assessment, information on obstetrical history (e.g. gravidity, parity, birth injuries), prior/present diseases of the genitourinary system (e.g. minor surgeries, incontinence, recurrent genital infections, symptomatic genital dryness), topic genital medication (e.g. vaginal suppository), point in time of last menstrual bleeding as well as latter gynecological examination, was obtained. Postmenopausal status was defined as cessation of menstrual bleeding for at least 12 months. Furthermore, behavioral aspects were surveyed including measures of intimate hygiene (e.g. tampon, waxing, depilation, shaving), contraception, sexual life (point in time of last penetrative sexual intercourse/masturbation, use of an object or lubricant of last penetrative intercourse, frequency of sexual intercourse), genital piercings (participant as well as sexual partner) and sports (e.g.horseback riding, cycling).

Clinical assessment including photo documentation was accomplished by one sole examiner, who was trained by forensic and gynecological physicians in visual inspection, colposcopy and toluidine-blue application prior to initiation of the investigation. Examination was accomplished in modified lithotomy position. Only the external genitals were assessed, slightly spreading and retracting labia majora. Colposcopy was accomplished with an ATMOS i View 31 (ATMOS MedizinTechnik GmbH, Lenzkirch, Germany) colposcope system, using × 4 magnification, including an integrated digital camera, Sony Alpha 5000 (Sony Corporation, Tokyo, Japan). After macroscopic examination and annotation of findings colposcopy and photo documentation using a measuring tape were conducted. Findings were noted again. Then, toluidine blue was applied on external genitals and consecutively removed with 1% acetic acid. Colposcopy and photo documentation were repeated, and findings again noted. In line with previous publications the following categories of injuries were assessed as follows [4, 6, 7]:

- Bruise: Located discoloration of intact epidermis due to an extravasation of blood.
- Abrasion: Areas of superficial epidermal disruption.
- Laceration: Discontinuity of epidermis and dermis.

### Statistics

All data was stored in a Microsoft Excel sheet. After a thorough check for false data entry, the data was imported into SAS® (release 9.4, SAS Institute Inc., Cary, NC, USA). For normally distributed quantitative variables mean and standard deviation were calculated. For ordinally scaled data and quantitative discrete data, median values together with minimum and maximum are given. For qualitative data, absolute and relative frequencies are presented.

In order to compare two mean values a 2-sample t-test was performed for data approximately normally distributed. Otherwise a Mann Whitney U-test has been used instead. The association between two qualitative factors was investigated with Fisher’s exact test. A test result with *p* < 0.05 was considered as statistically significant.

## Results

### Study population

A total of 200 participants were recruited; three questionnaires were returned incomplete and photo documentation of 6 women was rated as qualitatively insufficient. Hence, 191 observations were statistically assessed.

The mean age of the study population was 41 (standard deviation ± 13) years. Table 1 summarizes demographic data and gynecologic history; characteristics of sexual history are depicted in Table 2. A total of n = 10 (5%) women did not have sexual intercourse before being examined, n = 108 (57%) did have consensual sex more than 7 days before examination. The remaining 38% reported to have had sex within the last week.

**Table 1:** Demographic data and gynaecological history

Number of participants (*n* = 191)		
	n	*%*
Demographic data		
*Age category*		
16- 20 yrs	6	3
21- 30 yrs	39	20
31- 40 yrs	47	25
41- 50 yrs	62	33
51- 60 yrs	25	13
61- 70 yrs	8	4
71- 79 yrs	4	2
BMI category		
17- 18,4 kg/ m^2^	4	2
18,5- 24,9 kg/ m^2^	101	53
25- 29,9 kg/ m^2^	51	27
30- 34,9 kg/ m^2^	23	12
35- 39,9 kg/ m^2^	5	3
≥ 40 kg/ m^2^	6	3
No data	1	1
Marital status		
Married	90	47
Divorced	9	5
Widowed	8	4
Single	37	19
In a permanent relationship	47	25
Gynaecological history		
Menopausal status		
Premenopausal	151	79
Postmenopausal	38	20
No data	2	1
Obstetrical history		
Pragnancy in history		
None	74	39
≥ 1	117	61
Births in history		
None	93	49
≥ 1	98	51
Vaginal delivery in history	75	39
History of birth injury (vaginal/ perineal tears)	53	28
Time since last gynaecological examination		
≤ 2 d	27	14
3- 7d	19	10
> 7d	144	75
No data	1	1
Recurrent gynaecological symptoms in history		
Itching	13	7
Pain	11	6
Inflammation	10	5
Dryness	29	15
Skin alterations	1	1
None	127	67
Topical estrogen medication	8	4

**Table 2:** Characteristics of sexual history and potential influencing factors on genital trauma

Number of participants (*n* = 191)		
	*n*	*%*
Contraception		
Condom	44	23
Oral	29	15
Intrauterine contraceptive	14	7
Other	8	4
None	96	50
Time past since last vaginal intercourse		
≤ 2d	28	15
3- 7d	45	24
> 7d	108	57
None	10	5
Use of lubricant	6	3
Use of object (vibrator e.g.)	7	4
Painfull intercourse	40	21
Reported bleeding after intercourse	8	4
Reported lesion after intercourse	2	1
Frequency of sexual intercourse		
Once per month or less	39	20
Once per month to once per week	81	42
More often than once per week	42	22
None	29	15
Time past since last masturbation		
≤ 2d	13	7
3- 7d	22	12
> 7d	73	38
None	83	44
Intimate care		
Use of soaped water/ hand	53	28
Use of water/ towel or sponge	37	19
Use of water/ hand	101	53
Intimate hair removal method		
Use of wax	4	2
Use of hair removal cream	3	2
Shaving	160	84
Others	5	3
None	19	10
Sanitary products used during period		
Tampons	67	35
Pads	38	20
Others	53	28
None	33	17
Intimate piercing	5	3
History of cycling in the last 2 days	29	15
History of injuries in the genital area		
During shaving	6	3
Other cause	3	2

### Genital injury prevalence

Macroscopically a total of 9 women (5%) were found to have at least one genital lesion, two (1%) had more than one injury. A total of 11 lesions (n = 10 lacerations, n = 1 abrasion) were identified.

Using colposcopic examination only, 17 women (9%) were found to have at least one genital lesion. Seven (4%) of these had more than one lesion and a total of 25 lesions (laceration, abrasion, bruise) were categorized. After toluidine blue dye, 55 women (28%) had at least one lesion, of these 14 (7%) had more than one and a total of 73 lesions were recorded respectively. Considering postmenopausal women only, the injury prevalence was found to be substantially higher (42%, 16/38) compared to premenopausal individuals (24%, 36/151).

### Injury characteristics

Using solely colposcopic magnification, of the 17 women with a lesion, 12 (71%) had at least one lesion affecting the posterior fourchette, 6 (35%) with lesions affecting the perineum or the labia minora. No further site was found to be injured.

**Fig. 1:**
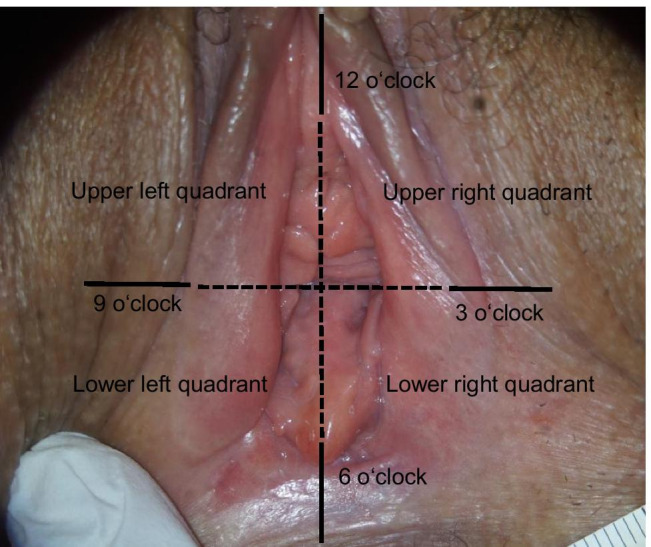
Subdivision of the outer genitals into quadrants

Subdividing the vaginal introitus into quadrants (see Fig. 1), the most common injury localization was the before mentioned “6 o’ clock position” (56%, 14/25), followed by the lower right quadrant (24%, 6/25) and the lower left quadrant (20%, 5/25). No lesions were found in the upper quadrants.

After toluidine blue dye 63% of genital trauma was found at the “6 o’clock position” (n = 46/73), 16% in the lower right (n = 12/73) and 15% in the lower left quadrant (n = 11/73) respectively. Additionally, one lesion was found in the upper right (1%; n = 1/73) and three (4%; n = 3/73) in the upper left quadrant. Table 3 summarizes injury characteristics. In our collective, regardless of the application of toluidine blue dye, a vertically aligned laceration was the most frequent type of injury observed.

**Table 3:** Injury characteristics

		Colposcopy		Colposcopy after toluidine blue dye	
		(*n* = 25)		(*n* = 73)	
		*n*	*%*	*n*	*%*
Injury type					
Laceration		20	80	45	62
Abrasio		5	20	28	38
Bruise		0	0	0	0
Alignement of the lacerations (n = 20/ 45)					
Vertical		13	65	36	80
Horizontal		2	10	2	4
Diagonal		5	25	7	16
Extension of lesions					
< 5 mm		16	64	56	77
5- 10 mm		9	36	15	21
> 10 mm		0	0	2	3
Marge of lesions					
Sharp		13	52	47	64
Blurred		12	48	26	36
Depth of lesions					
Superficial		24	96	72	99
Deep		1	4	1	1

### Factors influencing injury prevalence

We identified several factors correlating with the prevalence of genital injuries. As expected, the menopausal status appeared to have a significant impact (*p* = 0.0165), as well as vaginal dryness (*p* = 0.0228), usage of topical vaginal medication (*p* = 0.0369) and dyspareunia (*p* = 0.0234). Furthermore, genital injury was also found to inversely correlate with the frequency of intercourse (*p* = 0.0022). Hence, lower genital injury rates were found in women with a higher intercourse frequency. Sub-classifying the entire collective into pre- and postmenopausal women we found this observation persistent for the premenopausal group only (*p* = 0.0052). As explained below, it has to be stressed that the majority of women (57%) in our collective did not have intercourse within seven days before examination. The remaining factors assessed, which were shown to have no influence on injury prevalence, are summarized in Table 4.

**Table 4:** Insignificant influencing factors

		*p*-Value^b^
*Demographic data*		
Age		0,33^a^
Marital status		0,57
*Gynaecological history*		
Gravidity		0,27^c^
Parity		0,75^c^
Vaginal delivery in history		0,63^c^
Cesarean section in history		0,98^c^
History of birth injury (vaginal/ perineal tears)		0,55
Time since last gynaecological examination		0,58
History of recurrent itching		0,06
History of recurrent inflammation		0,47
Intimate care		0,71
Intimate hair removal method		0,25
Sanitary products used during period		0,84
Contraceptive method		0,19
*Sexual history*		
Masturbation		0,23
Penetrative consensual intercourse		0,09
Use of lubricant/ object		0,23
Intimate piercing		0,33
History of cycling in the past 2 days		0,08

^a^TTEST Procedure

^b^Fisher's exact Test if not differnetly indicated

^c^Wilcoxon Two-Sample Test

### “U “-shaped staining of the introitus after toluidine blue dye

**Fig. 2:**
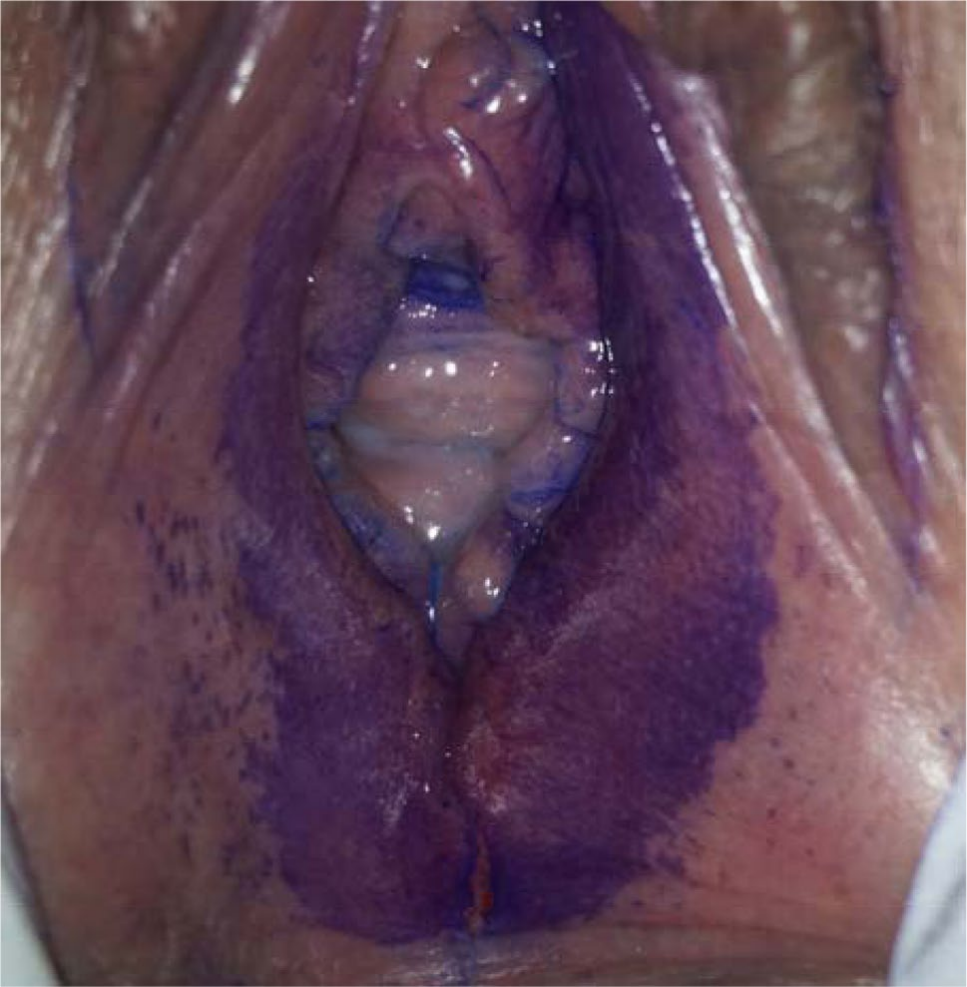
U-shaped staining of the introitus after toluidine blue dye

Performing the toluidine blue dye, we observed a “U”-shaped (see Fig. 2) staining in some women: this staining is strictly delimited and superficial, reaching from the posterior fourchette to the posterior half of the hymen as well as to the inner area of the labia minora. The anterior part of the vaginal introitus remains free of coloration. Analyzing data, we found this staining to correlate significantly with history of sexual intercourse (*p* = 0.0001). This staining was not detected in any participants without a history of vaginal intercourse. Of all women who reported to have had consenting intercourse, this staining was observed in 29% if intercourse took place within the past 2 days, and in 51% if intercourse took place within the last 3 to 7 days.

## Discussion

To the best of our knowledge and in analogy with a review of the literature by Astrup et al., there is a total of 9 key publications assessing genital injuries after consenting sexual intercourse [5]. While 7 investigations were comparative, contrasting genital trauma after consensual versus non-consensual intercourse, two studies focused on changes in genital injury patterns after consensual sex over the course of time. These investigations have in common that only premenopausal women were included. However, substantial differences were made concerning trauma classifications with some authors assessing lacerations only [8, 9], while others distinguished between bruise, abrasion and lacerations [3, 10–12] or utilized the TEARS (partially modified) classification [4, 6, 7].

Also, the method of examination in the existing literature differs substantially, with some investigations using macroscopic examination only [4, 6]. Others implemented macroscopic examination with toluidine dye 8, 9], colposcopy and toluidine dye [3, 7, 10, 11], or colposcopy and acetic acid [12] respectively. Due to these blatant methodical differences comparison of findings with differentiation of congruities has to be undertaken with caution.

### Prevalence

The first publications on genital trauma after consensual sexual intercourse were published by Laumer and McCauley et al. in the 1980s [8, 9]. Both investigations were prospective, recruiting women presenting with gynecological symptoms after consenting sexual intercourse, which has to be considered as a potential bias. While the number of patients include n the studies (22 and 48 patients with an injury prevalence of 5 and 10% respectively) were rather small, the authors assessed genital trauma using toluidine blue dye, focusing on lacerations only.

Slaughter et al. were the first to use a colposcope and toluidine dye enhancement to analyze genital trauma in rape victims using the TEARS classification as stated above [3]. Findings were compared with a consensual sex population consisting of 75 women (two minors) with a majority of women (n = 48) who were at first evaluated as rape victims but who later admitted having had consensual intercourse. While results due to the latter aspect have to be interpreted with caution, authors found an injury prevalence of 11%.

McLean et al. assessed 68 women presenting for a routine cervical PAP-smear after consenting intercourse macroscopically, without using either a colposcope or dye. Injuries, categorized as bruise, abrasion and/or laceration, were observed in 5.9% [6].

Similarly, Lincoln et al. examined a total of 81 women presenting for routine cervical screening or with sexual health concerns after consensual sex macroscopically [4]. Injuries, also defined as bruise, abrasion and/or laceration, were found in 9.9%.

The above stated investigations were comparative investigations that recruited women after consensual and non-consensual penetrative intercourse had occurred [3, 4, 6, 8, 9]. Other authors chose to recruit women prospectively, hence before scheduled consensual intercourse [7, 10–12]. In a comparative study Anderson examined, using a colposcope and toluidine dye, 46 women within 24 h after consensual intercourse differentiating trauma according to a modified TEARS classification (excluding swelling and redness) [10]. Authors found a genital injury prevalence of 30%. Analogously, Zink et al. examined 120 female volunteers detecting genital injuries in 55% [11].

Fraser et al. assessed 107 sexually active women two or three times over a 4- 6 month period using a consistent technique with colposcopic magnification and acetic acid [12]. Authors looked for changes in the vaginal and cervical appearance (TEARS with additional categories microtrauma and mucosal changes), which might be related to sexual intercourse or other environmental factors and found genital injuries in 25.2% within 24 h after sexual intercourse.

Finally, Astrup et al. examined a total of 98 women within 48 h after consensual intercourse and found genital injuries (bruise, abrasion, laceration) in 52% using colposcopy and toluidine dye [8].

In the above stated review of the literature it is worth noting that if examination takes places after intercourse, prevalence ranges from 4 to 11%, while prevalence is much higher (25 to 55%) if clinical assessment is accomplished before scheduled intercourse [5]. Prevalence of genital injury in our collective was 28% (including postmenopausal women; 24% for premenopausal women only). Reviewing the literature, it becomes obvious that the diagnostic methods used, definitions of injury, and the time of recruitment have an impact on injury prevalence. Of the cited investigations, the study designs (prospective design, usage of a colposcope with toluidine dye, classification of injury) by Astrup and Anderson et al. are best comparable to our data [7, 10]. However, it is important to consider in this context that 5% of women in our collective did not have intercourse at all before being examined while 57% of participants did not have consensual sex within the past 7 days before examination. Taking into account that the examined genital areas also differ slightly, as well as the above explained restrictions (especially the time of recruitment and the size of our study collective), a prevalence of approximately 25 to 30% seems to be realistic.

### Factors influencing injury prevalence

In the existing literature potential risk factors for genital trauma after consenting intercourse, analogously to injury prevalence, have to be considered with caution. However, reviewing the existing literature some findings of the above stated investigations are congruent. While existing data concerning skin color, penetrative use of finger(s) during intercourse and gynecological disease as potential influencing factors are controversial, findings are consistent concerning age, parity, use of lubricants and of tampons as well as contraceptives as the prevalence of injuries did not correlate with these factors [5]. Furthermore, besides timing of clinical examination as explained above (before versus after intercourse), Fraser et al. identified smoking status to correlate with injury prevalence: if women smoked prevalence was higher [12].

Our investigation additionally assessed postmenopausal women in this context and as expected menopause status had a significant impact on injury prevalence (*p* = 0.0165): at least one injury was observed in 42.1% of postmenopausal versus 23.8% of premenopausal women. There exists some information about genital trauma after non-consensual intercourse [3, 13–18]. Although most authors defined menopause status depending on age and not on menstruation, which has to be considered critically, there is high consistency that a postmenopause status influences genital trauma significantly. It has to be stressed that numeric age in our collective was not identified as a risk factor, which is consistent with the findings of other authors [4, 6, 7, 19]. Regardless of menopause status, vaginal dryness, dyspareunia and lower frequency of vaginal intercourse could be identified as further influencing factors in our study population. These factors are plausible, but have not been reported before. This circumstance is surprising, as vaginal dryness, which is known to correlate with dyspareunia, is a frequent symptom reported by approximately 15% of premenopausal and 57% of postmenopausal women [20–22]. Furthermore, vaginal dryness is arguably one of the major reasons for the increased prevalence of genital trauma in rape victims.

Interestingly, an increasing frequency of vaginal intercourse correlated inversely with genital injury. To what extent this finding is affected by an increased libido with corresponding lubrication is debatable. Furthermore, the majority of women (57%) in our collective did not have intercourse within seven days before examination. Fourteen percent stated they had had sex within 2 days and 24% within the last 3 to 7 days before being examined. In contrast to the findings of previous investigations, the prevalence of injury did not correlate with time passed since last intercourse: Anderson et al. found a significant reduction of injury size within 72 h after consensual intercourse. Astrup et al. found a “median survival time” of genital lesions after consenting sex of 24 h using the naked eye, 40 h using solely the colposcope, and 80 h using colposcope magnification and toluidine dye. What has to be considered in this context is the fact that in both investigations recruitment took place before examination and authors examined women only up to 3 (Anderson et al.) or 7 (Astrup et al.) days respectively [7, 23]. Our data, based on a distinct study design, should be considered as “real world data” concerning genital injury, including postmenopausal women with a demonstrably higher prevalence of genital lesions. Interestingly, of (57%) women who reported to not have had sexual intercourse within the past 7 days, 6 (6%) had evidence of genital injury macroscopically, 9 (8%) using colposcopic magnification and 38 (35%) using colposcopy with toluidine blue dye. Furthermore, we could observe a “U”-shaped coloration via toluidine dye of the introitus if women did have penetrative sex before examination (*p* = 0.0001). To the best of our knowledge this finding has not been described before and might be the result of blue coloration due to superficial epithelium desquamation. Future investigations assessing genital injury should focus on this observation.

In accordance with previous investigations potential influencing factors (see Table 4) such as age, parity, use of lubricants, vibrators and of tampons as well as contraceptives had no impact. Also, intimate care, method of pubic hear removal, masturbation, genital piercing and sports such as horseback riding and cycling, which to the best of our knowledge have not been investigated before, had no impact on injury prevalence either.

### Injury type and location of injury

In total, we detected 25 lesions (n = 17 women: 20 lacerations, 5 abrasions) colposcopically and 73 (n = 55 women: 45 lacerations, 28 abrasions) using colposcope and toluidine dye. Bruises were not found. These findings are in accordance with previous investigations, as lacerations were found to be the most common genital injury after consensual intercourse [4, 7–11, 19, 24]. Also concerning abrasions as second most frequent genital injury our results are consistent with existing publications [10, 11, 24].

In previous investigations all types of injury were observed, but despite methodical differences findings are highly consistent as most authors identified one single vertically aligned laceration affecting the posterior fourchette as the most frequent major type of injury [5]. Our results are consistent with these findings as 17 (9%) of women had injuries detected colposcopically and 55 (28%) using toluidine dye additionally. Of these 17 women, 12 (71%) had at least one lesion affecting the posterior fourchette, 6 (35%) involving the perineum or the labia minora respectively. No further locations were observed. In 59% (n = 10) of women with genital trauma using colposcope magnification and in 58% (n = 32) using additional toluidine dye the above stated vertically aligned laceration was found. These findings align with the results by Astrup et al., as the authors found the vertical laceration at the “6 o’clock position” in 75% of women with genital injuries colposcopically and in 81% with additional toluidine dye [24]. Concerning further genital injuries published data are inconsistent. Astrup et al. found the labia minora as the second most frequent localization (23/31% of women with genital injuries using colposcope/ additional toluidine dye) [24]. While in our collective labial damage was only observed in one woman, other investigations found no labial trauma at all [3, 4, 23]. On the other hand, Slaughter et al. state injuries of the hymen as the second most frequent (n = 3/ 8) [3]. As in both of these investigations the above stated bias have to be kept in mind, injuries of the labia minora as well as the hymen seem to be rather uncommon. In our collective the second most frequent were further lesions of the introitus: using colposcopy only 4 of 17 women (24%), with additional toluidine dye 11 of 55 (20%) respectively, had an injury affecting the lower right quadrant.

Few investigations focused on vaginal and cervical trauma, observing no [3, 4, 24] or occasional lesions [6, 11] of these structures after consensual intercourse.

Subdividing the topography of the external genital into quadrants we found 94% of lesions located in the lower quadrants. Hence, this region should be of special focus with respect to future scientific and forensic investigations.

Referring to the objective of this investigation, the findings of which have to be considered physiological, one can summarize that lacerations and/or abrasions of the lower quadrants (primarily a vertical laceration at the “6 o’clock position”), especially in postmenopausal women CAN be physiological.

## Conclusion

With and without history of consensual intercourse, using colposcope and toluidine blue dye during examinations, the prevalence of genital injury seems to range between 25 to 30%. By far the most frequent lesion that was observed was a vertically aligned laceration affecting the posterior fourchette. Generally, the posterior boundaries of the vaginal introitus seem to be predominantly affected if consenting or no sexual intercourse occurred. Genital trauma affecting the anterior boundaries of the introitus has to be considered unusual after consensual intercourse. This circumstance as well as the identified risk factors, particularly postmenopausal status, should be taken into account in legal proceedings of rape victims.

## Key points

1. The frequency of diagnostic injury differed substantially depending on the examination technique used, ranging from 9% using colposcopic magnification only to 28% with the additional use of toluidine blue dye.
2. A vertical laceration affecting the posterior fourchette was the most frequent lesion detected (17%, n = 32).
3. Menopausal status seems to have significant impact on genital injury prevalence (*p* = 0.0165), as 42% (16/38) of postmenopausal compared to 24% (36/151) of premenopausal women had at least one genital lesion.
4. Performing the toluidine blue dye, we observed a “U”-shaped (see Fig. 2) staining in some women. Analyzing data, we found this staining to correlate significantly with a history of sexual intercourse (*p* = 0.0001). This staining was not detected in any participant without a history of vaginal intercourse.

## Data Availability

The datasets generated during this investigation are available from the corresponding author on reasonable request.
